# Bone Mineralization and Calcium Phosphorus Metabolism

**DOI:** 10.3390/nu13113692

**Published:** 2021-10-21

**Authors:** María Luz Couce, Miguel Saenz de Pipaon

**Affiliations:** 1 Department of Pediatrics, University Clinical Hospital of Santiago de Compostela, 15704 Santiago de Compostela, Spain; 2 IDIS-Health Research Institute of Santiago de Compostela, 15704 Santiago de Compostela, Spain; 3 CIBERER, Instituto Salud Carlos III, 28029 Madrid, Spain; 4 MetabERN, Via Pozzuolo, 330, 33100 Udine, Italy; 5 Faculty of Medicine, Santiago de Compostela University, 15704 Santiago de Compostela, Spain; 6 Department of Neonatology, Hospital Universitario La Paz, 28046 Madrid, Spain

The accretion of adequate mineral content is essential for normal bone mineralization. Mineral homeostasis requires a careful balance of the actions of parathyroid hormone, calcitonin, FGF23, and vitamin D on target organs (kidney, intestine, and bone). Dietary deficiencies, particularly during the pediatric growth stages, and some clinical conditions are at increased risk of mineral imbalance and metabolic bone disease [1,2,3,4,5,6]. However, there are still gaps in our knowledge regarding several aspects of mineral and vitamin needs, and in the screening practices of mineral deficiency under certain conditions, such as prematurity and metabolic disorders. The evaluation of premature infants at risk for osteomalacia generally includes serum screening with the measurement of total or ionized calcium, phosphorus, and alkaline phosphatase activity. It is very important to identify infants and children who are at risk to prevent or treat mineral homeostasis alterations.

This Special Issue of *Nutrients*, “Bone Mineralization and Calcium Phosphorus Metabolism” contains four original publications and two reviews investigating bone status in prematurity and in patients with inherited metabolic diseases, as well as the contribution of mineral intake to metabolic bone disease (MBD), which suggests the growth and extensive interest in research on this topic.

Three studies were published examining how the alterations in calcium phosphorus metabolism can be identified, prevented, and treated in very premature infants. Very low birthweight (VLBW) infants, i.e., those weighing <1500 g at birth, are at a higher risk of significant mineral deficiencies when compared to term infants. As in older children, osteomalacia/rickets is generally due to a deficient mineral supply or uptake. Avila et al. [7] reported the clinical factors associated with biochemical indicators of the MBD of infants born at ≤32 weeks of gestation, with a birthweight of ≤1500 g. The authors concluded that the incidence of MBD in very preterm infants remains notable, affecting more than 1 in 10 infants according to early biochemical criteria. Birthweight was the only independent risk factor for MBD, and red blood cell transfusion was an independent risk factor for high risk of MBD. Llorente Pelayo et al. [8] aimed to modify serum alkaline phosphatase (M-ALP) as a biomarker for MBD in preterm infants, and use ultrasound monitoring for the apparition of knee ossification centers as a marker of bone mineralization (particularly in preterm infants with cholestasis). The authors concluded that the dual approach, combining M-ALP measures with the ultrasound monitoring of ossification centers favors the optimization of MBD treatments. In one important additional study [9], Mihatsch et al. hypothesized that VLBW infants who were appropriate weight for their term-equivalent age (ATEA; defined by an SD weight score > −2 at 36 weeks according to Alexander growth charts, *n* = 39) have higher growth rates, lean body mass and fat mass, skeletal mineral deposition, and neurodevelopmental scores throughout the first three years of life than light for TEA (LTEA), defined by a weight standard deviation (SD) score of less than −2 SD at 36 weeks of gestation (*n* = 55). Growth, bone mineral density, body composition, and metabolic health outcome was evaluated in a prospective cohort of 94 very low birthweight infants whose in-hospital target macronutrient intake was within the recent European Society of Pediatric Gastroenterology, Hepatology and Nutrition (ESPGHAN) recommendations. Child dual-energy X-ray absorptiometry (DXA) scans were performed at 6, 12, 18, 24, and 36 months. All DXA scans were performed with the same device (Lunar-DPX-MD) and software (infant whole-body analysis, GE Healthcare, Chalfont Saint Giles, UK). The results demonstrate that, from six months of corrected age, and into the third year of life, gains in weight, length and head circumference, mid arm circumference, adiposity, fat free mass, and bone mineralization in preterm infants are lower than in-term infants, and influenced by nutritional status at discharge. The authors speculate that, in hospitals, VLBW infants may require a higher nutrient intake. Low birthweight preterm infants’ LTEA appear to especially benefit from targeted preventive interventions to improve growth, bone mineralization, and neurodevelopmental outcome, without increasing obesity risk.

Regarding inherited metabolic disease studies, de Castro et al. [10] evaluated bone mineral density and its relation to food intake in children with inborn errors of intermediary metabolism (IEiM). Patients with IEiM undergoing dietary treatment, especially those with amino acid and carbohydrate metabolism disorders, present alterations in body composition, including a reduced height, a tendency towards overweight and obesity, and a reduced bone mineral density. Moderate to-vigorous physical activity was positively correlated with bone mineral density and muscle mass, suggesting that regular physical activity may play a key role in optimizing body composition in IEiM patients.

Two reviews investigating the impact of certain conditions on bone mineralization and the need for nutritional interventions to prevent alterations in bone metabolism were published. De Castro et al. [11], in a systematic review, reported that bone mineral density was lower in phenylketonuria (PKU) patients than in reference groups, but was within the normal range in most patients when expressed as z-score value. An imbalance between bone formation and bone resorption, favoring bone removal, was observed. Mihatsch et al. [12] reviewed the physiology of fetal mineral accretion and preterm infants’ mineral absorption, and concluded that calcium and phosphorus requirements depend on weight gain. They additionally concluded that current calcium and phosphorus recommendations systematically underestimate the needs of preterm infants, given a target neonatal weight gain above 17 g/kg/day, particularly for Ca; therefore, a higher enteral fortifier/formula mineral content, or individual supplementation, is required.

The present Special Issue provides a summary of the progress on the topic of calcium phosphorus metabolism and its important role in bone mineralization in risk populations, which will be of interest from a clinical and public health perspective. Nevertheless, clinical studies with a longer follow-up are needed to understand mineral homeostasis alterations and establish recommendations to provide an adequate, balanced nutritional supply of energy, proteins, vitamins, calcium, phosphorus and other nutrients, to improve bone development and short- and long-term outcomes, including long-term bone health and fractures in later life.
